# Inhibition of Excessive Monoamine Oxidase A/B Activity Protects Against Stress-induced Neuronal Death in Huntington Disease

**DOI:** 10.1007/s12035-014-8974-4

**Published:** 2014-11-15

**Authors:** Jolene Ooi, Michael R. Hayden, Mahmoud A. Pouladi

**Affiliations:** 1 Translational Laboratory in Genetic Medicine (TLGM), Agency for Science, Technology and Research (A*STAR), 8A Biomedical Grove, Immunos, Level 5, Singapore, 138648 Singapore; 2 Centre for Molecular Medicine and Therapeutics, Child and Family Research Institute, University of British Columbia, Vancouver, British Columbia V5Z 4H4 Canada; 3 Department of Medicine, Yong Loo Lin School of Medicine, National University of Singapore, Singapore, 117597 Singapore

**Keywords:** Huntington’s disease, Striatal neural cells, Monoamine oxidase, Oxidative stress, Monoamine oxidase inhibitors, Human-induced pluripotent stem cells

## Abstract

Monoamine oxidases (MAO) are important components of the homeostatic machinery that maintains the levels of monoamine neurotransmitters, including dopamine, in balance. Given the imbalance in dopamine levels observed in Huntington disease (HD), the aim of this study was to examine MAO activity in a mouse striatal cell model of HD and in human neural cells differentiated from control and HD patient-derived induced pluripotent stem cell (hiPSC) lines. We show that mouse striatal neural cells expressing mutant huntingtin (HTT) exhibit increased MAO expression and activity. We demonstrate using luciferase promoter assays that the increased MAO expression reflects enhanced epigenetic activation in striatal neural cells expressing mutant HTT. Using cellular stress paradigms, we further demonstrate that the increase in MAO activity in mutant striatal neural cells is accompanied by enhanced susceptibility to oxidative stress and impaired viability. Treatment of mutant striatal neural cells with MAO inhibitors ameliorated oxidative stress and improved cellular viability. Finally, we demonstrate that human HD neural cells exhibit increased MAO-A and MAO-B expression and activity. Altogether, this study demonstrates abnormal MAO expression and activity and suggests a potential use for MAO inhibitors in HD.

**Electronic supplementary material:**

The online version of this article (doi:10.1007/s12035-014-8974-4) contains supplementary material, which is available to authorized users.

## Introduction

Huntington disease (HD) is an autosomal dominant neurodegenerative disorder that has no known cure. It is caused by an expansion of a CAG trinucleotide repeat within exon 1 of the *huntingtin* (*HTT*) gene, where the onset of motor symptoms and severity of neuropathology are dependent on the size of the trinucleotide expansion [1, 2]. The clinical profile of HD includes motor, cognitive, and psychiatric dysfunction. Pathologically, HD is characterized by preferential loss of neurons in the striatum and deep layers of the cortex.

Molecular assessments during disease progression show discrepancies in neurotransmitter levels [3, 4]. Dopamine, a monoamine, is one such neurotransmitter. Dopamine homeostasis and dopamine-regulated pathways are altered in HD mouse models [5, 6]. In the R6/1 mouse model of HD, the levels of striatal dopamine and its metabolite homovanillic acid are significantly reduced [7]. Dopamine turnover is also altered in R6/1 mice [7]. In R6/2 mice, significant deficits are observed in extracellular and tissue levels of striatal dopamine and in D1-class dopamine receptor signaling [5, 8–10]. Similarly, baseline and evoked extracellular levels of striatal dopamine are reduced in YAC128 HD mice [5, 10, 11]. In pre-symptomatic HD gene carriers, a progressive loss of dopamine D1 and D2 receptor (D1R and D2R) binding is observed by PET imaging [12–15]. Furthermore, in post mortem HD brains, there is a reduced number of striatal D1R- and D2R-positive cells as assessed by messenger RNA (mRNA) detection [1, 16], and the levels of striatal dopamine are also significantly reduced [3, 17].

One avenue of processing dopamine is through the activity of monoamine oxidase (MAO) enzymes. MAOs are flavin-containing enzymes located on the mitochondrial outer membrane which oxidatively deaminate monoamines, generating hydrogen peroxide as a by-product [5, 18]. Present as two isozymes, MAO-A and MAO-B are encoded by separate genes adjacent to each other on the short arm of the X chromosome. They also have identical exon-intron structural organization suggesting that they were derived as a result of ancestral gene duplication [5, 18]. The two isozymes share 70 % similarity in amino acid sequence. Dopamine is a common substrate for both isozymes but MAO-A has a higher affinity toward serotonin, norepinephrine, and epinephrine [5, 19, 20]. Although MAO proteins are found throughout the brain, MAO activity varies considerably from region to region, with the caudate showing the highest levels of activity [12, 21].

Abnormal MAO-A and MAO-B activity has been implicated in various mental and neurodegenerative disorders [16, 21], and pharmacological inhibitors of MAO have been shown to be of clinical benefit [17, 21]. Given the marked deficits in dopamine levels and signaling in HD, the aim of this study was to examine MAO activity in HD patient and rodent-derived cellular systems.

## Materials and Methods

### Cell Culture and Transfections

ST*Hdh* ^Q7/Q7^ and ST*Hdh* ^Q111/Q111^ cells were generated as described by [18, 22]. The cells were grown in Dulbecco’s modified Eagle’s medium (DMEM; Gibco) with 10 % fetal bovine serum (FBS; HyClone) and 5 mM glutamine (Gibco). To transfect the cells, cells were seeded and grown until 90 % confluency before FuGENE® 6 (Promega) was used according to the manufacturer’s instructions. In brief, a DNA/FuGENE ratio of 1:3 was used and the cells were incubated with the mix over 24 h before media was changed.

Human dermal fibroblasts and HD patient-derived induced pluripotent stem cell (hiPSC) lines from HD patients and controls were obtained from Coriell. The sample IDs are listed in Table 1. The dermal fibroblast cells were grown in α-MEM (HyClone) with 10 % FBS (HyClone) and 5 mM glutamine (Gibco). The hiPSC lines were grown as described in the “Derivation of Neural Progenitor Cells” section.

**Table 1:** Information table showing Coriell cell repository IDs, genotypes, gender, race, age and CAG size

Sample ID	Description	Gender	Race	Age	CAG size
ND29971	HD gene-negative (control)	F	Caucasian	61	20/19
ND30014	HD gene-negative (control)	F	Unknown	52	21/18
ND31008	HD gene-negative (control)	F	Unknown	–	17/17
ND31845	HD gene-negative (control)	F	Caucasian	73	19/18
ND32603	HD gene-negative (control)	F	Unknown	62	19/17
ND33391	HD gene-negative (control)	F	Unknown	–	19/17
ND30625	HD gene-negative (control)	M	Caucasian	76	18/17
ND31037	HD gene-negative (control)	M	Caucasian	30	19/17
ND30015	HD gene-positive	F	Unknown	28	41/21
ND30047	HD gene-positive	F	Caucasian	23	41/19
ND30259	HD gene-positive	F	Unknown	74	38/21
ND33392	HD gene-positive	F	Unknown	29	57/17
ND33947	HD gene-positive	F	Unknown	71	40/18
ND29970	HD gene-positive	M	Caucasian	65	40/17
ND30626	HD gene-positive	M	Caucasian	62	41/17
ND31551	HD gene-positive	M	Unknown	19	39/18
GM2183	Fibroblast line used to generate CAG33 iPSC lines	F	Caucasian	21	33/18
GM9197	Fibroblast line used to generate CAG180 iPSC lines	M	Caucasian	6	180/18

### Serum Starvation and Drug Treatment

To starve ST*Hdh* ^Q7/Q7^ and ST*Hdh* ^Q111/Q111^ cells of serum, cells were seeded onto appropriate tissue culture plates and grown until confluent. The cells were washed once with PBS before medium without serum (DMEM with 5 mM glutamine) was added. If MAO inhibitors were included into the experiment, the drugs were added upon the start of 24-h serum starvation.

### Derivation of Neural Progenitor Cells

CAG33 and CAG180 hiPSCs employed in this study were described by the HD iPSC Consortium, 2012 [23]. Neural progenitor cells (NPCs) were derived using the protocol described in Li et al. [24]. In brief, CAG33 and CAG180 hiPSCs at about 20 % confluence were treated with N_2_B_27_ media (DMEM/F12: neurobasal media (1:1), 1× N_2,_ 1× B_27_, 1× pen/strep/glutamine, 10 ng/mL hLIF (Millipore), 5 μg/mL BSA) containing 3 μM CHIR99021 (Tocris), 2 μM SB431542 (Tocris), and 0.1 μM compound E for 7 days. The culture was then split 1:3 for the next six passages using Accutase without compound E on Matrigel-coated plates.

### RNA Isolation and Quantitative PCR

RNA from cells was extracted using RNeasy mini kit (QIAGEN) according to the manufacturer’s instructions. To generate complementary DNA (cDNA), Superscript® II Reverse Transcription Kit (Life Technologies) was used. A total of 20 μL of cDNA was generated per 1 μg of RNA. To perform quantitative PCR (qPCR), cDNA was diluted 10-fold and 2 μL was used per qPCR reaction. To complete the reaction volume, 0.67 mM primers and SYBR® Select Master Mix (Life Technologies) were added. The primers used were as follows:

- mMAO-A-F: 5′ GCCCAGTATCACAGGCCAC 3′,
- mMAO-A-R: 5′ GTCCCACATAAGCTCCACCA 3′
- mMAO-B-F: 5′ ATGAGCAACAAAAGCGATGTGA 3′,
- mMAO-B-R: 5′ TTCTAATTGTGTAAGTCCTGCCT 3′,
- mActin-F: 5′ GGCTGTATTCCCCTCCATCG 3′,
- mActin-F: 5′ CCAGTTGGTAACAATGCCATGT 3′,
- hMAO-A-F: 5′ TTCAGGACTATCTGCTGCCAA 3′,
- hMAO-A-R: 5′ GGTCCCACATAAGCTCCACC 3′,
- hMAO-B-F: 5′ GCTCTCTGGTTCCTGTGGTATGTG 3′,
- hMAO-B-R: 5′ TCCGCTCACTCACTTGACCAGATC 3′,
- hActin-F: 5′ GTCTTCCCCTCCATCGTG 3′,
- hActin-R: 5′ GATGGGGTACTTCAGGGTGA 3′
- hOCT4-F: 5′ TCTTTCCACCAGGCCCCCGGCTC 3′
- hOCT4-R: 3′ TGCGGGCGGACATGGGGAGATCC 3′
- hNESTIN-F: 5′ GAAACAGCCATAGAGGGCAAA 3′
- hNESTIN-R: 5′ TGGTTTTCCAGAGTCTTCAGTGA 3′
- hPAX6-F: 5′ GTGTCTACCAACCAATTCCACAAC 3′
- hPAX6-R: 5′ CCCAACATGGAGCCAGATG 3′
- hSOX1-F: 5′ CAGTACAGCCCCATCTCCAAC 3′
- hSOX1-R: 5′ GCGGGCAAGTACATGCTGA 3′

### Immunoblotting

ST*Hdh* ^Q7/Q7^ and ST*Hdh* ^Q111/Q111^ cells were pelleted and lysed with homogenization buffer (50 mM Tris HCl pH 8.0, 150 mM NaCl, 1 % IGEPAL, 1 mM PMSF, 5 μM z-VAD and 1× complete protease inhibitor cocktail (Roche)). The cell pellets were resuspended in the homogenization buffer and incubated on ice for 20 min. The lysates were centrifuged at 4 °C at 13,300 rpm for 15 min and the supernatant was transferred to a new tube. The protein concentration was measured using the Bradford Assay (Bio-Rad). Five micrograms of protein was used for each immunoblotting analysis. Protein lysates were separated on a 7 % acrylamide gel and transferred onto a nitrocellulose membrane. Blots were incubated overnight at 4 °C. The antibodies used were anti-calnexin (Sigma C4731 1:2000 dilution), anti-MAO-A (Abcam ab126751 1:1000 dilution), anti-MAO-B (GeneTex GTX105790 1:1000 dilution). Alexa Fluor 680 goat anti-mouse and goat anti-rabbit (Molecular Probes) were used as secondary antibodies (1:3000 dilution). Li-Cor Odyssey Infrared Imaging system was used for detection and quantification.

### Luciferase Assays

To perform the luciferase assays, manufacturer’s instructions were followed. The protocols used are briefly described below:

### Dual-Glo Luciferase Assay System

In brief, the Dual-Glo Luciferase Assay System (Promega) was used to assay for MAO-A and MAO-B transcriptional activity. Cells were lysed using 5× passive lysis buffer, while shaking at room temperature for 15 min. Fifty microliters of the lysate was transferred into an opaque 96-well plate before equal volume of Luciferin Detection Reagent was added. After incubation at room temperature for 20 min, the signal was measured using FLUOstar Omega (BMG Labtech). To quench the firefly luciferase signal, Stop and Glo buffer was added and Renilla luciferase signal was measured.

### CellTiter-Glo®

Cells were washed with PBS after drug treatment and 50 μL of fresh media was added to each well. Equal volume of CellTiter-Glo buffer (Promega) was added to each well and the plate was left to shake at room temperature for 10 min before readings were taken.

### MTT Assay

After treatment, cells in a 96-well plate were washed once with 1× PBS and incubated with 100 μL of 1 mg/mL MTT (Sigma) in DMEM and incubated at 37 °C for 4 h. The cells were subsequently washed with 1× PBS before adding 100 μL of DMSO. MTT readings were taken at absorbance 570 nm.

### GSH/GSSG-Glo™ Assay

Cells were washed after drug treatment and 50 μL of either total or oxidized glutathione reagent (Promega) was added to each well. After 5 min of shaking, equal volume of luciferin-generation reagent was added and incubated at room temperature for 30 min. Lastly, 100 μL of luciferin detection reagent was added and luminescence was measured.

### MAO-Glo Assay System

Protein lysates were diluted to 0.5 mg/mL using lysis buffer. Twenty-five microliters of the diluted lysate was incubated with 25 μL of MAO substrate solution (1:250 dilution of provided MAO substrate to measure MAO-B activity and 1:25 of provided MAO substrate to measure MAO-A activity) (Promega) for 2 h at room temperature. Fifty microliters of luciferin detection reagent was then added and luminescence was measured.

### ATP/ADP Measurements

To measure ATP/ADP ratios, ATP/ADP assay kit (Abcam) was used according to the manufacturer’s instructions. In brief, upon treatment in a 96-well plate, nucleotides were released using 100 μL of nucleotide-releasing buffer, before 50 μL of each well was taken to measure its ATP/ADP ratio.

### Immunostaining

hiPSCs and NPCs were seeded on Matrigel-coated 13 mm coverslips in 24-well plates. Upon reaching the desired confluency, the wells were washed with 1× PBS and fixed with 4 % formaldehyde in PBS at room temperature for 30 min. The coverslips were washed three times for 5 min each using wash buffer (PBS with 0.3 % Triton X-100 and 2 % goat serum) and blocked with blocking buffer (PBS with 3 % Triton X-100 and 3 % goat serum) at room temperature for 1 h. For staining, the following antibodies and concentrations were used: anti-OCT3/4 (sc-9081; Santa Cruz) 1:100; anti-NESTIN (MAB5326; Millipore) 1:500. Antibodies were diluted in wash buffer and incubated at 4 °C overnight. The cells were then washed three times for 5 min each using wash buffer and stained with secondary antibodies (Alexa Fluor, Life Technologies) at a dilution of 1:200. After three times of 5-min washes, the cells were stained with DAPI and mounted onto microscope slides using ProLong® Gold Antifade Mountant (Life Technologies).

### Statistical Analysis

Data are expressed as means ± SEM. Statistical significance was ascertained by one- or two-way ANOVA with appropriate post hoc testing or by Student’s *t* test. Where testing of normality was possible, the D’Agostino and Pearson omnibus normality test was used to assess distribution of values. Differences were considered statistically significant when *p* < 0.05.

## Results

### MAO-A/B Activity is Elevated in ST*Hdh*^Q111/Q111^ Cells

**Fig. 1:**
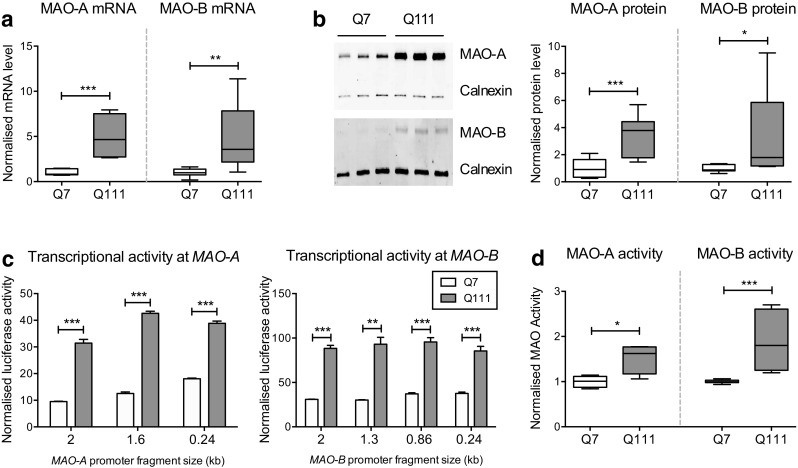
ST*Hdh* ^Q111/Q111^ cells exhibit increased MAO-A/B expression and activity when compared to ST*Hdh* ^Q7/Q7^ cells. **a** Quantitative RT-PCR shows increased MAO-A and MAO-B expression in ST*Hdh* ^Q111/Q111^ cells when compared to ST*Hdh* ^Q7/Q7^ cells. Values shown were normalized to ST*Hdh* ^Q7/Q7^ cells; *n* = 8; **b** A representative Western blot (*left*) showing increased MAO-A and MAO-B expression in ST*Hdh* ^Q111/Q111^ cells when compared to ST*Hdh* ^Q7/Q7^ cells. (*right*) Quantification and normalization of the band intensities demonstrate that MAO-A and MAO-B are expressed to a greater extent in ST*Hdh* ^Q111/Q111^ cells than ST*Hdh* ^Q7/Q7^ cells. Values shown were normalized to ST*Hdh* ^Q7/Q7^ cells; *n* = 9. **c** Transcriptional activities at MAO-A and MAO-B promoter regions are elevated in ST*Hdh* ^Q111/Q111^ cells compared to ST*Hdh* ^Q7/Q7^ cells. ST*Hdh* ^Q111/Q111^ cells exhibited increased luciferase activity across all promoter lengths, suggesting that transcriptional activity at MAO-A and MAO-B promoter regions are elevated in ST*Hdh* ^Q111/Q111^ cells, compared to ST*Hdh* ^Q7/Q7^ cells; *n* = 3. ***p < 0.001 when compared to all the respective controls. **d** MAO-A and MAO-B activities are elevated in ST*Hdh* ^Q111/Q111^ cells. MAO-A and MAO-B activities were measured using MAO-Glo™ Assay System (Promega). ST*Hdh* ^Q111/Q111^ cells displayed higher activity when compared to ST*Hdh* ^Q7/Q7^ cells; *n* = 9. *Error bars* in the *bar chart* represent standard error of the mean, whereas the *whiskers* on the *box plots* represent minimum and maximum values. Q7 and Q111 refer to ST*Hdh* ^Q7/Q7^ and ST*Hdh* ^Q111/Q111^ cells, respectively. **a**–**d** *p < 0.05; **p < 0.01; ***p < 0.001 by unpaired two-tailed *t* test

To determine MAO-A/B expression patterns in a HD model, we chose to study a set of well-characterized, immortalized mouse striatal cell lines: the ST*Hdh* ^Q7/Q7^ and ST*Hdh* ^Q111/Q111^ cells. ST*Hdh* ^Q7/Q7^ and ST*Hdh* ^Q111/Q111^ cells were derived from E14 striatal primordia of mice expressing 7 and 111 CAG repeats within exon 1 of the *Hdh* locus, respectively [18, 25–27]. Assessment of MAO-A/B mRNA levels in the two cell lines showed that MAO-A and MAO-B transcripts were expressed at levels approximately fivefold higher in ST*Hdh* ^Q111/Q111^ than ST*Hdh* ^Q7/Q7^ cells (unpaired two-tailed *t* test; *p* < 0.0001 for MAO-A, *p* = 0.0052 for MAO-B) (Fig. 1a). Consistent with the differences in transcript levels, quantification of protein levels showed that MAO-A and MAO-B protein levels were fourfold and twofold higher in ST*Hdh* ^Q111/Q111^ than ST*Hdh* ^Q7/Q7^ cells, respectively (unpaired two-tailed *t* test; *p* = 0.0003 for MAO-A, *p* = 0.0321 for MAO-B) (Fig. 1b).

Luciferase reporters that were previously established [17, 19, 20] were employed to examine transcriptional activities at MAO-A and MAO-B promoter loci (Fig. 1c). Regions spanning 0.24 to 2 kb upstream of the MAO-A/B transcriptional start site have been described as being critical to promote transcription [21, 28]. We show that MAO-A/B promoter fragments of up to 2 kb were transcriptionally active in both ST*Hdh* ^Q7/Q7^ and ST*Hdh* ^Q111/Q111^ cells and, importantly, that the transcriptional activity at the MAO-A/B promoter loci was significantly increased in ST*Hdh* ^Q111/Q111^ cells compared to ST*Hdh* ^Q7/Q7^ cells (unpaired two-tailed *t* test; for MAO-A *p* < 0.001 compared to respective ST*Hdh* ^Q7/Q7^ controls; for MAO-B *p* < 0.001 for 0.24-, 0.86-, and 2-kb fragments, and *p* < 0.01 for 1.3-kb fragment compared to respective ST*Hdh* ^Q7/Q7^ controls) (Fig. 1c). To determine the functional significance of the differences in MAO expression, MAO activity was measured in both cell lines. In line with the expression and transcriptional activity reporter data, MAO-A and MAO-B activity were elevated by approximately 1.5-fold and 2-fold in ST*Hdh* ^Q111/Q111^ when compared to ST*Hdh* ^Q7/Q7^ cells, respectively, (unpaired two-tailed *t* test; *p* < 0.05 for MAO-A, and *p* < 0.001 for MAO-B) (Fig. 1d).

### Stress-induced Elevation in MAO Activity Contributes to Reduced Viability in ST*Hdh*^Q111/Q111^ Cells

**Fig. 2:**
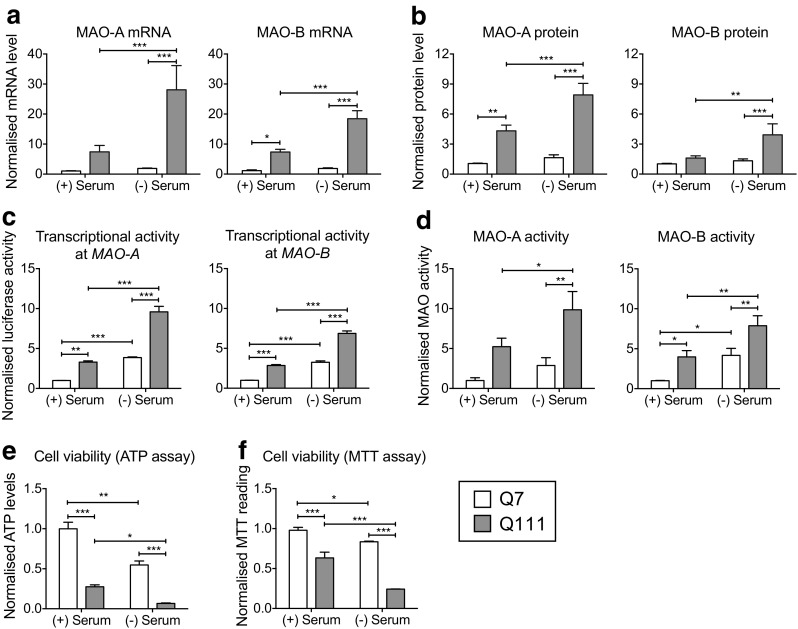
Serum deprivation increases MAO expression and activity in ST*Hdh* ^Q111/Q111^ cells. **a** Quantitative RT-PCR shows increased MAO-A and MAO-B expression in ST*Hdh* ^Q111/Q111^ cells upon serum starvation for 24 h. Values shown were normalized to untreated ST*Hdh* ^Q7/Q7^ cells; *n* = 5; (two-way ANOVA; for MAO-A genotype: *F*(1,33) = 25.70, *p* < 0.0001; treatment: *F*(1,33) = 11.17, *p* = 0.0021; genotype × treatment: *F*(1,33) = 9.440, *p* = 0.0042; for MAO-B genotype: *F*(1,19) = 42.76, *p* < 0.0001; treatment: *F*(1,19) = 11.30, *p* = 0.0033; genotype × treatment: *F*(1,19) = 8.695, *p* = 0.0084; Fisher’s LSD post hoc); **b** MAO-A and MAO-B protein levels increase upon 24-h serum starvation in ST*Hdh* ^Q111/Q111^ cells. Values shown were normalized to untreated ST*Hdh* ^Q7/Q7^ cells; *n* = 9; (two-way ANOVA; for MAO-A genotype: *F*(1,32) = 54.08, *p* < 0.0001; treatment: *F*(1,32) = 10.15, *p* = 0.0032; genotype × treatment: *F*(1,32) = 5.210, *p* = 0.0292; for MAO-B genotype: *F*(1,26) = 11.97, *p* < 0.0019; treatment: *F*(1,26) = 8.032, *p* = 0.0088; genotype × treatment: *F*(1,26) = 4.644, *p* = 0.0406; Fisher’s LSD post hoc). **c** Transcriptional activity at MAO-A and MAO-B promoters increases upon serum starvation. Transcriptional activity at the MAO-A and MAO-B promoter regions (2-kb promoter fragment) increased upon serum starvation in ST*Hdh* ^Q7/Q7^ and ST*Hdh* ^Q111/Q111^ cells; *n* = 3; (two-way ANOVA; for MAO-A genotype: *F*(1,8) = 126.6, *p* < 0.0001; treatment: *F*(1,8) = 164.4, *p* < 0.0001; genotype × treatment: *F*(1,8) = 22.93, *p* = 0.0014; for MAO-B genotype: *F*(1,8) = 208.7, *p* < 0.0001; treatment: *F*(1,8) = 275.0, *p* < 0.0001; genotype × treatment: *F*(1,8) = 21.60, *p* = 0.0016; Fisher’s LSD post hoc) **d** MAO-A and MAO-B activities are enhanced upon serum starvation. MAO-A and MAO-B activities were measured using MAO-Glo™ Assay System (Promega). ST*Hdh* ^Q7/Q7^ cells and ST*Hdh* ^Q111/Q111^ cells displayed higher activity upon 24-h serum starvation, with the increase being significantly greater in ST*Hdh* ^Q111/Q111^ cells; *n* = 11; (two-way ANOVA; for MAO-A genotype: *F*(1,15) = 15.97, *p* = 0.0012; treatment: *F*(1,15) = 5.348, *p* = 0.0354; genotype × treatment: *F*(1,15) = 0.9548, *p* = 0.3440; for MAO-B genotype: *F*(1,46) = 12.90, *p* = 0.0008; treatment: *F*(1,46) = 14.42, *p* < 0.0004; genotype × treatment: *F*(1,46) = 0.1515, *p* = 0.6989; Fisher’s LSD post hoc). **e** Serum withdrawal in ST*Hdh* ^Q7/Q7^ cells and ST*Hdh* ^Q111/Q111^ cells results in decreased ATP levels. ATP levels were measured using CellTiter-Glo® upon 24-h serum starvation; *n* = 4; (two-way ANOVA; genotype: *F*(1,12) = 137.4, *p* < 0.0001; treatment: *F*(1,12) = 44.80, *p* < 0.0001; genotype × treatment: *F*(1,12) = 9.469, *p* = 0.0096; Fisher’s LSD post hoc). **f** Serum withdrawal results in greater reduction in cell viability in ST*Hdh* ^Q111/Q111^ cells as assessed by the MTT assay. ST*Hdh* ^Q7/Q7^ and ST*Hdh* ^Q111/Q111^ cells were serum starved for 24 h before incubation with 1 mg/mL MTT for 4 h; *n* = 4; (two-way ANOVA; genotype: *F*(1,12) = 148.5, *p* < 0.0001; treatment: *F*(1,12) = 44.85, *p* < 0.0001; genotype × treatment: *F*(1,12) = 6.167, *p* = 0.0288; Fisher’s LSD post hoc). *Error bars* in the *bar chart* represent standard error of the mean, whereas the *whiskers* on the *box plots* represent minimum and maximum values. Q7 and Q111 refer to ST*Hdh* ^Q7/Q7^ and ST*Hdh* ^Q111/Q111^ cells, respectively. *p < 0.05; **p < 0.01; ***p < 0.001

To examine the effect of differential MAO activity in ST*Hdh* ^Q7/Q7^ and ST*Hdh* ^Q111/Q111^ cells, we employed a cellular stress paradigm to magnify effects induced by disparities in MAO activity. We chose the widely used serum starvation cellular stress paradigm which has previously been shown to increase MAO expression, and sought to determine the effect of serum starvation in ST*Hdh* ^Q7/Q7^ and ST*Hdh* ^Q111/Q111^ cells on MAO activity and cell viability [22, 29]. Serum starvation of ST*Hdh* ^Q7/Q7^ and ST*Hdh* ^Q111/Q111^ cells for 24 h led to increased MAO-A/B mRNA (Fig. 2a), and in particular, in ST*Hdh* ^Q111/Q111^ cells compared to ST*Hdh* ^Q7/Q7^ (two-way ANOVA; for MAO-A genotype: *F*(1,33) = 25.70, *p* < 0.0001; treatment: *F*(1,33) = 11.17, *p* = 0.0021; genotype × treatment: *F*(1,33) = 9.440, *p* = 0.0042; for MAO-B genotype: *F*(1,19) = 42.76, *p* < 0.0001; treatment: *F*(1,19) = 11.30, *p* = 0.0033; genotype × treatment: *F*(1,19) = 8.695, *p* = 0.0084; Fisher’s least significant difference (LSD) post hoc). Similarly, MAO-A/B protein levels were significantly higher in ST*Hdh* ^Q111/Q111^ cells compared to ST*Hdh* ^Q7/Q7^ following serum starvation (Fig. 2b) (two-way ANOVA; for MAO-A genotype: *F*(1,32) = 54.08, *p* < 0.0001; treatment: *F*(1,32) = 10.15, *p* = 0.0032; genotype × treatment: *F*(1,32) = 5.210, *p* = 0.0292; for MAO-B genotype: *F*(1,26) = 11.97, *p* < 0.0019; treatment: *F*(1,26) = 8.032, *p* = 0.0088; genotype × treatment: *F*(1,26) = 4.644, *p* = 0.0406; Fisher’s LSD post hoc). From an epigenetic perspective, MAO-A/B promoter regions (2 kb fragment) were more transcriptionally active upon serum deprivation, with significantly higher transcriptional activity in ST*Hdh* ^Q111/Q111^ compared to ST*Hdh* ^Q7/Q7^ cells (Fig. 2c) (two-way ANOVA; for MAO-A genotype: *F*(1,8) = 126.6, *p* < 0.0001; treatment: *F*(1,8) = 164.4, *p* < 0.0001; genotype × treatment: *F*(1,8) = 22.93, *p* = 0.0014; for MAO-B genotype: *F*(1,8) = 208.7, *p* < 0.0001; treatment: *F*(1,8) = 275.0, *p* < 0.0001; genotype × treatment: *F*(1,8) = 21.60, *p* = 0.0016; Fisher’s LSD post hoc). Similar results were obtained with the shorter MAO-A/B promoter fragments (data not shown). Consistent with these results, MAO-A and MAO-B activity were significantly elevated after 24 h of serum starvation in both ST*Hdh* ^Q7/Q7^ and ST*Hdh* ^Q111/Q111^ cells, but with a significantly greater increase in the ST*Hdh* ^Q111/Q111^ cells (Fig. 2d) (two-way ANOVA; for MAO-A genotype: *F*(1,15) = 15.97, *p* = 0.0012; treatment: *F*(1,15) = 5.348, *p* = 0.0354; genotype × treatment: *F*(1,15) = 0.9548, *p* = 0.3440; for MAO-B genotype: *F*(1,46) = 12.90, *p* = 0.0008; treatment: *F*(1,46) = 14.42, *p* < 0.0004; genotype × treatment: *F*(1,46) = 0.1515, *p* = 0.6989; Fisher’s LSD post hoc).

Serum starvation is a widely used method to induce cell death in many cellular systems, including the ST*Hdh* ^Q7/Q7^ and ST*Hdh* ^Q111/Q111^ cells [5, 25–27]. We deprived ST*Hdh* ^Q7/Q7^ and ST*Hdh* ^Q111/Q111^ cells of serum over 24 h and showed that ATP levels were reduced upon serum withdrawal (Fig. 2e), suggesting that serum starvation led to reduced cell viability. Interestingly, ST*Hdh* ^Q111/Q111^ cells have inherently lower ATP levels compared to ST*Hdh* ^Q7/Q7^ cells. In addition, serum starvation in ST*Hdh* ^Q7/Q7^ cells led to a twofold reduction in cell viability, whereas serum starvation in ST*Hdh* ^Q111/Q111^ cells resulted in a fourfold reduction in cell viability, suggesting that ST*Hdh* ^Q111/Q111^ cells were more susceptible to serum withdrawal-induced reduction in cell viability (Fig. 2e) (two-way ANOVA; genotype: *F*(1,12) = 137.4, *p* < 0.0001; treatment: *F*(1,12) = 44.80, *p* < 0.0001; genotype × treatment: *F*(1,12) = 9.469, *p* = 0.0096; Fisher’s LSD post hoc). Similar results were obtained with the MTT cell viability assay (Fig. 2f) (two-way ANOVA; genotype: *F*(1,12) = 148.5, *p* < 0.0001; treatment: *F*(1,12) = 44.85, *p* < 0.0001; genotype × treatment: *F*(1,12) = 6.167, *p* = 0.0288; Fisher’s LSD post hoc).

### Reduced Viability Results from Excessive MAO A/B Activity in ST*Hdh*^Q111/Q111^ Cells and Is Associated with Increased Oxidative Stress

**Fig. 3:**
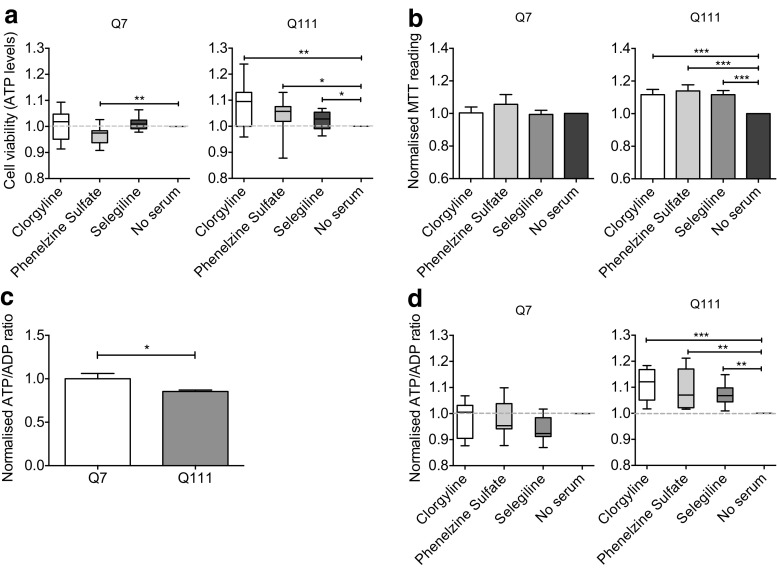
Elevated MAO-A/B activity compromises ATP metabolism and reduces cell viability in serum-deprived ST*Hdh* ^Q111/Q111^ cells. **a** MAO inhibition rescues reduced ATP levels in serum-deprived ST*Hdh* ^Q111/Q111^ cells. ST*Hdh* ^Q7/Q7^ (*left*) and ST*Hdh* ^Q111/Q111^ (*right*) cells were treated with 1 μM of MAO inhibitors upon the start of serum starvation over 24 h. ATP levels were measured using CellTiter-Glo®. Values shown were normalized to no serum controls. No significant improvements were observed in ST*Hdh* ^Q7/Q7^ cells but inhibition of MAO in ST*Hdh* ^Q111/Q111^ cells resulted in an increase of ATP levels; *n* = 12; (one-way ANOVA, *p* = 0.0168; individual treatment versus no serum control comparisons by one-tailed *t* test). **b** MAO inhibition rescues serum deprivation induced cell death in ST*Hdh* ^Q111/Q111^ cells. ST*Hdh* ^Q7/Q7^ (*left*) and ST*Hdh* ^Q111/Q111^ (*right*) cells were treated with 1 μM of MAO inhibitors upon the start of serum starvation over 24 h before incubation with 1 mg/mL MTT for 4 h. Values shown was normalized to no serum controls. Inhibition of MAO in ST*Hdh* ^Q7/Q7^ cells had no significant effect on cell death but inhibition of MAO in ST*Hdh* ^Q111/Q111^ cells resulted in decreased cell death; *n* = 21; (one-way ANOVA for ST*Hdh* ^Q7/Q7^ *p* = 0.6178; one-way ANOVA for ST*Hdh* ^Q111/Q111^ *p* = 0.0027; individual treatment versus no serum control comparisons by one-tailed *t* test). **c** MAO inhibition rescues reduced ATP/ADP ratios in serum-deprived ST*Hdh* ^Q111/Q111^ cells. ST*Hdh* ^Q7/Q7^ (*left*) and ST*Hdh* ^Q111/Q111^ (*right*) cells were treated with 1 μM of MAO inhibitors upon the start of serum starvation over 24 h. ATP/ADP ratios were measured using an ATP/ADP assay kit. Values shown were normalized to no serum controls. A significant improvement was only observed in ST*Hdh* ^Q111/Q111^ cells where inhibition of MAO resulted in an increase in ATP/ADP ratio; *n* = 6; (*p* < 0.05 by one-tailed *t* test); **d** ST*Hdh* ^Q111/Q111^ exhibit lower ATP/ADP ratios compared to ST*Hdh* ^Q7/Q7^ cells. ATP/ADP ratios were measured in cells grown in normal culture conditions, in the presence of serum. Values shown were normalized to ST*Hdh* ^Q7/Q7^ cells; *n* = 3; (one-way ANOVA; for ST*Hdh* ^Q7/Q7^ *p* = 0.1677; for ST*Hdh* ^Q111/Q111^ *p* = 0.0123, Fisher’s LSD post hoc); *Error bars* in the *bar chart* represent standard error of the mean, whereas the *whiskers* on the *box plots* represent minimum and maximum values. Q7 and Q111 refer to ST*Hdh* ^Q7/Q7^ and ST*Hdh* ^Q111/Q111^ cells, respectively. *p < 0.05; **p < 0.01; ***p < 0.001

To determine if increased MAO activity plays a role in the reduction of cell viability, we introduced MAO inhibitors upon the start of serum deprivation. Three MAO inhibitors were used: (a) clorgyline, an irreversible MAO-A-specific inhibitor, (b) phenelzine sulfate, an irreversible non-selective MAO inhibitor, and (c) selegiline, an irreversible MAO-B specific inhibitor [17, 30]. The presence of MAO inhibitors at 1 μM led to improvements in cell viability as assessed by CellTiter-Glo assay following serum starvation in ST*Hdh* ^Q111/Q111^ but not ST*Hdh* ^Q7/Q7^ cells, suggesting that increased MAO activity in ST*Hdh* ^Q111/Q111^ cells contributes to the serum starvation-induced reduction in cell viability (Fig. 3a) (one-way ANOVA, *p* = 0.0168; individual treatment versus “no serum” control comparisons by one-tailed *t* test). No improvements were observed with 0.1 or 0.01 μM of MAO inhibitors (data not shown). As MAO activity in ST*Hdh* ^Q7/Q7^ cells was inherently lower, inhibition of MAO activity did not yield a significant rescue of the serum starvation-induced reduction in cell viability in these cells. Similar results were obtained using the MTT assay of cell viability (Fig. 3b) (one-way ANOVA for ST*Hdh* ^Q7/Q7^ *p* = 0.6178; one-way ANOVA for ST*Hdh* ^Q111/Q111^ *p* = 0.0027; individual treatment versus no serum control comparisons by one-tailed *t* test).

The CAG tract expansion in *HTT* has been previously shown to result in compromised cellular metabolism and to lead to a reduced cellular ATP/ADP ratio [31, 32]. This phenomenon has been observed in a number of human and rodent HD cellular systems including HD patient lymphoblasts, hiPSC-derived neural cells, and the ST*Hdh* ^Q111/Q111^ striatal cells [23, 31, 32]. Consistent with previous studies, we observed a significantly lower ATP/ADP ratio in ST*Hdh* ^Q111/Q111^ compared to ST*Hdh* ^Q7/Q7^ (Fig. 3c) (*p* < 0.05 by one-tailed *t* test). To examine the impact of MAO inhibition on this property, we measured the ATP/ADP ratio in serum-starved ST*Hdh* ^Q7/Q7^ and ST*Hdh* ^Q111/Q111^ cells at baseline and following treatment with MAO inhibitors. While we observed no effect of MAO inhibition on the ATP/ADP ratio in ST*Hdh* ^Q7/Q7^ cells (Fig. 3d) (one-way ANOVA; *p* = 0.1677), treatment with MAO inhibitors resulted in a significant improvement of the ATP/ADP ratio in ST*Hdh* ^Q111/Q111^ cells (Fig. 3d) (one-way ANOVA; *p* = 0.0123; Fisher’s LSD post hoc).

**Fig. 4:**
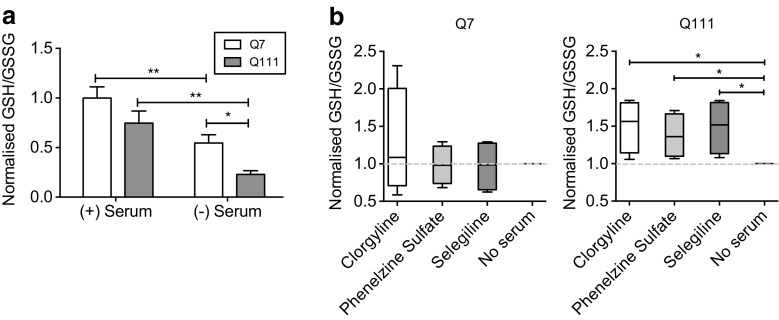
Reduced viability resulting from excessive MAO-A/B activity is associated with increased oxidative stress. **a** Oxidative stress increases upon serum starvation in ST*Hdh* ^Q7/Q7^ and ST*Hdh* ^Q111/Q111^ cells. GSH/GSSG levels are a surrogate measure of oxidative stress. GSH/GSSG levels of ST*Hdh* ^Q7/Q7^ and ST*Hdh* ^Q111/Q111^ cells were measured in serum and in 24-h serum-starved conditions. Values shown were normalized to ST*Hdh* ^Q7/Q7^ cells. Upon 24-h serum starvation, both ST*Hdh* ^Q7/Q7^ and ST*Hdh* ^Q111/Q111^ cells displayed a decrease in GSH/GSSG levels. Furthermore, GSH/GSSG levels were significantly lower in ST*Hdh* ^Q111/Q111^ compared to ST*Hdh* ^Q7/Q7^ cells following serum starvation; *n* = 4; (two-way ANOVA; genotype: *F*(1,12) = 9.086, *p* = 0.0108; treatment: *F*(1,12) = 26.37, *p* = 0.0002; genotype × treatment: *F*(1,12) = 0.7452, *p* = 0.7452; Fisher’s LSD post hoc) **b** MAO inhibition reduces oxidative stress levels in serum-deprived ST*Hdh* ^Q111/Q111^ cells. GSH/GSSG levels were measured in 24-h serum-starved ST*Hdh* ^Q7/Q7^ (*left*) and ST*Hdh* ^Q111/Q111^ (*right*) cells in the presence of varying concentrations of MAO inhibitors. MAO inhibitors were added upon the start of serum starvation. Values shown were normalized to no serum controls. 1 μM of the inhibitors were capable of increasing GSH/GSSG levels, hence, reduce oxidative stress in ST*Hdh* ^Q111/Q111^ cells but not ST*Hdh* ^Q7/Q7^ cells; *n* = 4; (individual treatment versus no serum control comparisons by unpaired two-tailed *t* test). The *whiskers* on the *box plots* represent minimum and maximum values. Q7 and Q111 refer to ST*Hdh* ^Q7/Q7^ and ST*Hdh* ^Q111/Q111^ cells, respectively. *p < 0.05; **p < 0.01; ***p < 0.001

As the deamination activity of MAO isozymes triggers oxidative reactions, we quantified oxidized (GSSG) and reduced forms of glutathione (GSH) in both ST*Hdh* ^Q7/Q7^ and ST*Hdh* ^Q111/Q111^ cells upon serum starvation as a measure of oxidative stress. We demonstrated that oxidative stress increased upon the removal of serum (Fig. 4a) (two-way ANOVA; genotype: *F*(1,12) = 9.086, *p* = 0.0108; treatment: *F*(1,12) = 26.37, *p* = 0.0002; genotype × treatment: *F*(1,12) = 0.7452, *p* = 0.7452; Fisher’s LSD post hoc), reflecting a trend where both GSH/GSSG ratios and cell viability decrease upon serum withdrawal (Fig. 2e, f). This suggests that elevated oxidative stress may contribute to lower cell viability. ST*Hdh* ^Q111/Q111^ cells experienced a higher oxidative stress burden upon serum deprivation, evident from a 3.2-fold decrease in GSH/GSSG ratio upon serum starvation, compared to a 1.8-fold decrease in ST*Hdh* ^Q7/Q7^ cells. This was consistent with the reduction in cell viability observed in Fig. 2e, where serum deprivation resulted in fourfold reduction in viability in ST*Hdh* ^Q111/Q111^ cells, compared to a twofold reduction in ST*Hdh* ^Q7/Q7^ cells.

To understand if MAO-A/B isozymes play a role in oxidative stress, thereby influencing cell viability, we treated ST*Hdh* ^Q111/Q111^ and ST*Hdh* ^Q7/Q7^ cells with MAO inhibitors upon removal of serum and measured GSH/GSSG ratios (Fig. 4b). Similar to cell viability measurements, the presence of MAO inhibitors at 1 μM preferentially reduced oxidative stress in ST*Hdh* ^Q111/Q111^, but not ST*Hdh* ^Q7/Q7^ cells, reinforcing the notion that elevated expression and activity of MAO-A/B in ST*Hdh* ^Q111/Q111^ cells contributes to increased oxidative stress, and thus impaired cell viability.

### MAO-A/B Expression and Activity in HD Patient Fibroblasts and hiPSC-derived Neural Cells

**Fig. 5:**
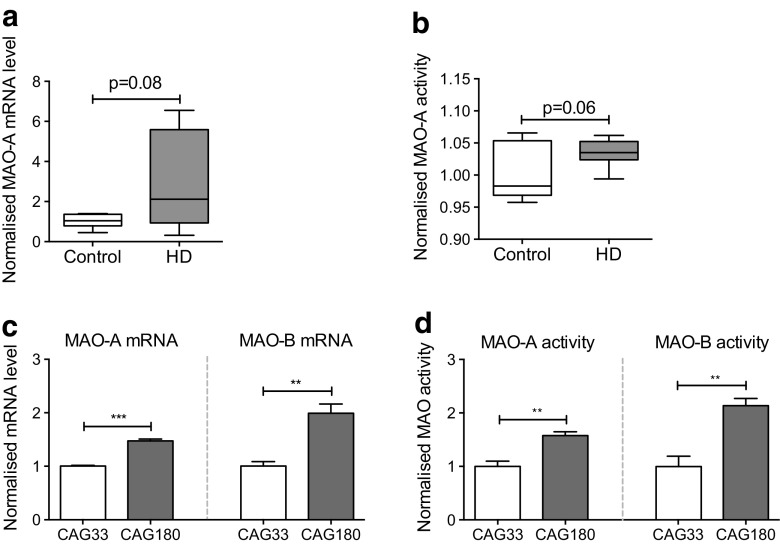
MAO-A/B expression and activity are increased in human HD neural cells. **a** Eight HD and eight control fibroblasts were analyzed for MAO-A mRNA levels. Values shown were normalized to control fibroblasts. Without segregation of age and gender, HD patient fibroblasts display a trend toward increased MAO-A expression compared to their control counterparts; *n* = 8; (unpaired two-tailed *t* test; *p* = 0.08). **b** Using MAO-Glo™ Assay System, MAO-A activities in the fibroblast lines were tested. Compared against the control fibroblasts, HD patient fibroblasts exhibited a trend toward increased MAO-A activity; *n* = 8; (unpaired two-tailed *t* test; *p* = 0.06); **c** Quantitative RT-PCR shows increased MAO-A and MAO-B expression in CAG180 NPCs when compared to CAG33 NPCs. Values shown were normalized to CAG33 NPCs. CAG33 and CAG180 hiPSCs were described by the HD iPSC Consortium, 2012 [23] and were differentiated into NPCs according to the protocol described by Li et al., 2011 [24]; *n* = 3; (unpaired two-tailed *t* test; *p* < 0.001 for MAO-A mRNA and *p* = 0.007 for MAO-B mRNA). **d** MAO-A and MAO-B activities are elevated in CAG180 NPCs. MAO-A and MAO-B activities were measured using MAO-Glo™ Assay System (Promega). Values shown were normalized to CAG33 NPCs. CAG33 NPCs displayed higher activity when compared to CAG33 NPCs. *Error bars* in the *bar chart* represent standard error of the mean, whereas the whiskers on the box plots represent minimum and maximum values; *n* = 3-4; (unpaired two-tailed *t* test; *p* = 0.005 for MAO-A activity and *p* = 0.006 for MAO-B activity). **p < 0.01; ***p < 0.001

Of the two MAO isozymes, only MAO-A is expressed in fibroblasts. To assess our findings from a clinical perspective, we measured MAO-A expression and activity in eight HD patient and eight control fibroblasts. Females and males were represented in both the control and HD donor groups; six control females (five HD females) and two control males (three HD males). Taking the longer CAG polynucleotide repeat length of the two alleles into consideration, the control group carried an average high CAG allele length of 19 ± 1.2 CAG repeats whereas the HD group carried an average high CAG allele length of 42 ± 6.1 CAG repeats. The mean ages of the control and HD donors were 59 ± 16.6 and 46 ± 23.6, respectively. Gender and age-related changes of platelet MAO have been reported where MAO activity increases with age [28]. Despite a lower average age of the HD donors, it is evident that a trend presents where HD patient fibroblasts exhibit higher levels of MAO-A expression and activity, although this difference did not reach statistical significance (Fig. 5a–b) (unpaired two-tailed *t* test; *p* = 0.08 for MAO-A mRNA and *p* = 0.06 for MAO-A activity).

As many neuronal subtypes express both MAO-A and MAO-B, we sought to determine whether human HD neural cells exhibit elevated MAO levels and activity. To this end, we employed a well-established directed differentiation protocol [24] to obtain human neural progenitor cells (NPCs) from previously described HD hiPSC lines (Supplementary Figure 1) [23]. Both MAO-A and MAO-B mRNA levels were significantly elevated in the HD NPCs compared to control NPCs (Fig. 5c) (unpaired two-tailed *t* test; *p* < 0.001 for MAO-A mRNA and *p* = 0.007 for MAO-B mRNA). Similarly, MAO-A and MAO-B activity levels were elevated in HD NPCs compared to control NPCs (Fig. 5d) (unpaired two-tailed *t* test; *p* = 0.005 for MAO-A activity and *p* = 0.006 for MAO-B activity).

## Discussion

This study demonstrates that MAO-A/B expression and activity are elevated in the presence of an expanded polyglutamine tract in HTT. Increased MAO-A/B activity plays a role in increasing oxidative stress and increasing susceptibility to stress-induced impairment in cell viability. Although oxidative stress is only one contributing factor to cell death, it is the most pertinent in the context of MAO isozymes, given their roles in oxidative metabolism. Elevation of MAO activity was not confined to a cellular model as a similar trend was observed in HD patient iPSC-derived neural cells. Overall, this is the first study that looks at the effect of enhanced MAO activity using biochemical assays in a well-established striatal and neural cell populations. In addition, this platform allowed us to evaluate the effects of pharmacological inhibitors of MAO activity and show that MAO inhibition alleviated stress-induced impairment in cell viability in HD mutant cells.

There have been previous reports on the analysis of MAO expression and activity in HD patients. With the first report in 1978, studies have shown that there is elevated MAO-B activity in HD patient platelet populations [33–35]. Increased post mortem MAO-B activity in patients suffering from HD has also been shown [36–38]. It is important to note, however, that most of these studies were performed before the causative mutation in *HTT* was identified. As a result, these studies did not exclude patients that were HD phenocopies, and thus were unable to ascertain that the effect is HD-specific.

In contrast, this study employs genetically defined mouse and human neural cell populations, highlighting that elevation of MAO activity is intrinsic to the presence of the mutant *HTT* allele. Although a number of studies reported increased MAO-B activity in HD patients and cells, one study did not observe differences in MAO-B activity in platelets among 59 HD patients, 20 at-risk individuals, 29 control individuals with an HD family history, and 64 healthy controls [29]. This discrepancy in reported MAO activity in HD patients may reflect lack of characterization of the study cohort. Indeed, lifestyle habits, such as smoking, have been shown to influence MAO-B activity (reviewed in [5]). As the study cohort comprised a mixture of HD patients with undefined smoking habits and receiving various drug interventions, these variations could have obscured differences in MAO activity. In general, the findings from previous studies are consistent with our data where we show that MAO-A/B activity and expression are increased in human neural cells originating from HD patients iPSCs.

MAO isozymes play a major role in metabolizing biogenic and dietary amines, generating hydrogen peroxide as a by-product. It is conceivable that increased MAO activity in the presence of mutant HTT results in an accumulation of reactive oxygen species (ROS). In support of this notion, recent studies have reported that ST*Hdh* ^Q111/Q111^ cells exhibit increased mitochondrial ROS [30] and addition of a glutathione peroxidase (GPx) supplement results in the reduction of cell death [2, 39]. As heightened activity of MAO-A/B leads to an increase in ROS production, it is possible that MAO acts upstream of GPx to increase ROS production, thus contributing to cell death.

Analogous to our findings with MAO inhibitors, a recent study has demonstrated that knockdown of MAO-A expression leads to increased ATP levels [40]. MAO-A/B is located in the outer mitochondrial membrane and their activity leads to the production of metabolic by-products like H_2_O_2_. In HD mutant cells, increased MAO-A/B activity implicates an accumulation of ROS. Due to close proximity to the mitochondrial oxidative phosphorylation system, it is conceivable that electron flow through the respiratory chain may be impaired and ATP levels may be reduced by ROS, possibly contributing to the lower ATP/ADP ratios seen in HD mutant cells.

Several studies have reported deficits in dopamine levels and signaling in animal models and patients with HD (reviewed in [4, 6]). Although this study suggests that increased MAO expression and activity contribute to deficiencies in dopamine homeostasis in a HD mutant background, it is important to note that MAO isozymes metabolize other substrates. These include serotonin, epinephrine, norepinephrine, phenylethylamine, and benzylamine [6, 21]. As there is a dearth of studies measuring these neurotransmitter levels in HD, future studies should investigate whether other MAO-metabolized neurotransmitters are affected in a fashion similar to dopamine as well as the impact of MAO inhibitors on the levels of these neurotransmitters.

Despite the unclear link between the presence of mutant HTT and increased MAO transcriptional activity, there have been separate lines of evidence that may explain this phenomenon. HTT has been shown to regulate Sp1 transcription factor where mutant HTT forms an enhanced association with Sp1 [8–10, 41, 42]. As MAO-A/B are both transcriptionally regulated by Sp1 and Sp3 [10, 11, 21, 22], it is possible that close interaction between mutant HTT and Sp1 results in increased MAO expression. Sp1 also affects D2 receptor expression levels, perhaps perturbing the dopamine metabolic pathway, and indirectly resulting in increased MAO-A/B expression.

Although this study delineates the role of increased MAO activity in a HD mutant background, several questions remain. The present study was conducted mostly using a striatal cell line that carries 111 CAG repeats. The size of the CAG repeat length in *HTT* affects the age of onset of motor symptoms and severity of neuropathology [2, 13–15]; hence, it will be interesting to determine if there is a relationship between the CAG size and the magnitude of MAO activity. Although we demonstrate that human neural cells derived from HD patient iPSCs exhibit increased MAO activity, the next step would be to validate these findings in a larger cohort and to examine this phenomenon in other tissue types.

In summary, this study provides evidence that cellular HD models exhibit increased MAO expression and activity, highlighting the potential of MAO-A/B as pharmacological targets in HD. MAO-B inhibitors are currently employed in the clinical setting to treat Parkinsonian symptoms. In addition, MAO inhibitors have also been shown to possess anti-depressant properties. As the cardinal features of HD are cognitive, motor, and psychiatric deficits, MAO inhibitors represent potential candidates for therapeutic intervention in HD.

## Electronic Supplementary Material

Below is the link to the electronic supplementary material.

Supplementary Figure 1 Characterization of CAG33 and CAG180 NPCs. **a** Immunostaining of CAG33 and CAG180 NPCs shows an induction of early neuronal marker, Nestin. CAG33 and CAG180 hiPSCs were differentiation over 7 days, and passaged once before immunostaining. Expression of Nestin (green) indicates the acquisition of neuronal identity, whereas the loss of Oct3/4 (*red*) indicates the loss of pluripotency. The images were taken at ×20 magnification. Scale bar = 100 μM. **b** Quantitative RT-PCR shows increased neuronal markers Nestin, Pax6 and Sox1, and reduced pluripotency-associated markers Oct3/4. NPCs passaged once after 7 days of differentiation were analyzed for their gene expression profile. Successful neuronal differentiation was shown by upregulation of neuronal markers and down-regulation of pluripotency-associated markers. (Comparisons to respective undifferentiated hiPSC line by unpaired two-tailed *t* test);**p < 0.01; ***p < 0.001. (PDF 3416 kb)
