# Supplementary figures and images for: Inhibition of Excessive Monoamine Oxidase A/B Activity Protects Against Stress-induced Neuronal Death in Huntington Disease
Source: Mol Neurobiol. 2014 Nov 15;52(3):1850–61. doi: 10.1007/s12035-014-8974-4 (PMC4586002; doi:10.1007/s12035-014-8974-4)

Supplementary Figure 1

A

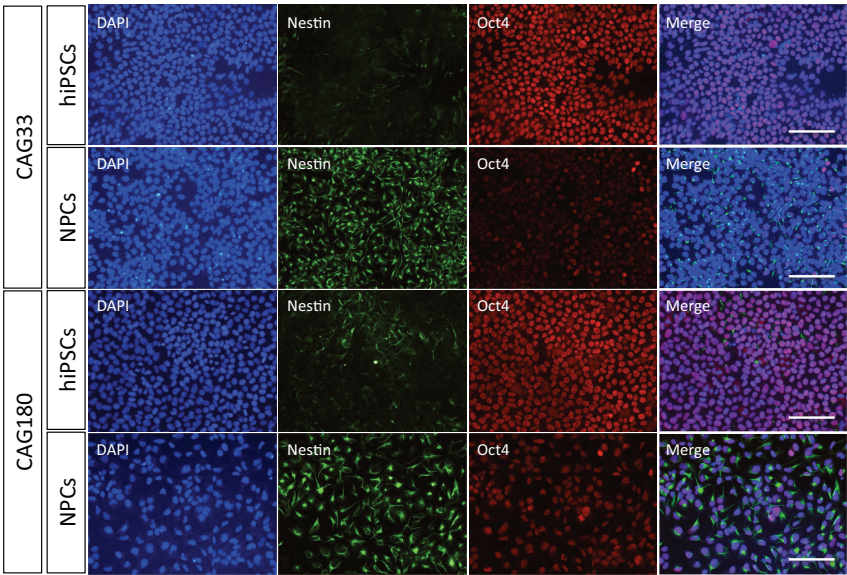

B

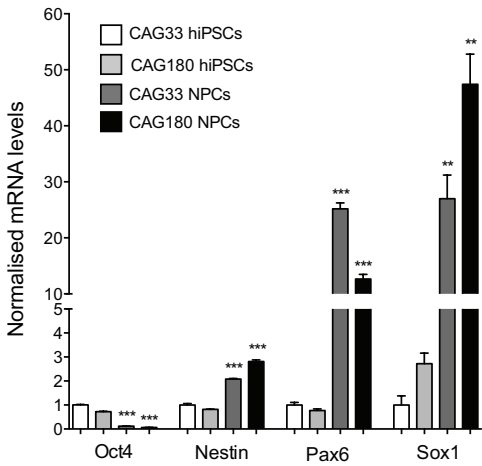

Supplement: Supplementary file 1 — Characterization of CAG33 and CAG180 NPCs. a Immunostaining of CAG33 and CAG180 NPCs shows an induction of early neuronal marker, Nestin. CAG33 and CAG180 hiPSCs were differentiation over 7 days, and passaged once before immunostaining. Expression of Nestin (green) indicates the acquisition of neuronal identity, whereas the loss of Oct3/4 (red) indicates the loss of pluripotency. The images were taken at ×20 magnification. Scale bar = 100 μM. b Quantitative RT-PCR shows increased neuronal markers Nestin, Pax6 and Sox1, and reduced pluripotency-associated markers Oct3/4. NPCs passaged once after 7 days of differentiation were analyzed for their gene expression profile. Successful neuronal differentiation was shown by upregulation of neuronal markers and down-regulation of pluripotency-associated markers. (Comparisons to respective undifferentiated hiPSC line by unpaired two-tailed t test);**p < 0.01; ***p < 0.001. (PDF 3416 kb) [file 12035_2014_8974_MOESM1_ESM.pdf]
